# Treatment effects of Chinese medicine (Yi-Qi-Qing-Jie herbal compound) combined with immunosuppression therapies in IgA nephropathy patients with high-risk of end-stage renal disease (TCM-WINE): study protocol for a randomized controlled trial

**DOI:** 10.1186/s13063-019-3989-9

**Published:** 2020-01-06

**Authors:** Shen Li, Jin-pu Li

**Affiliations:** Renal Division, Guang’anmen Hospital, China Academy of Chinese Medical Sciences, No. 5 Bei Xian Ge St. Xi Cheng District, Beijing, 10053 China

**Keywords:** IgA nephropathy, Immunosuppressive, High-risk IgAN, Yi-Qi-Qing-Jie formula, Traditional Chinese medicine

## Abstract

**Background:**

IgA nephropathy (IgAN) is the most common glomerular disease worldwide. It has a high incidence in Asians and is more likely to progress to end-stage renal disease (ESRD). For high-risk IgAN, which is clinically characterized by massive proteinuria and renal dysfunction, however, there has been no international consensus on treatment options. Compared with other developed countries, IgAN patients in China are often found to have severe kidney function loss at initial diagnosis. Yi-Qi-Qing-Jie formula (YQF; a compound recipe of Chinese medicinal herbs) has shown potential renal protection in our previous clinical studies. To further confirm the efficacy and safety of YQF in the treatment of high-risk IgAN, we have designed a prospective double-blind randomized placebo-controlled trial.

**Methods/design:**

The TCM-WINE study is a single-center, prospective, double-blind randomized placebo-controlled trial. We plan to randomize 60 participants with biopsy-proven IgAN to a YQF combined group (YQF compound combined with prednisolone, and cyclophosphamide if necessary) or an immunosuppression group (placebo-YQF combined with prednisolone, and cyclophosphamide if necessary). The two groups will enter a 48-week in-trial treatment phase and receive post-trial follow-up until study completion (3 years). All patients will receive optimal supportive care. The primary composite outcome is defined as the first occurrence of a 40% decrease in estimated glomerular filtration rate (eGFR) from the baseline lasting for 3 months, initiating continuous renal replacement treatment, or death due to chronic kidney disease (CKD) during the 3-year study phase. The secondary endpoint events are defined as the mean annual eGFR decline rate (eGFR slope, ml/min per 1.73 m^2^ per year), which is calculated by the eGFR regression curve for each eligible patient, and proteinuria remission (prescribed as proteinuria < 0.5 g/day) at weeks 24, 36, and 48 during the in-trial phase. The remission rate of symptoms and inflammation status will be evaluated at week 48. Safety monitoring and assessment will be undertaken during the study.

**Discussion:**

The TCM-WINE study will evaluate the effects and safety of YQF combined therapy compared with immunosuppression monotherapy on the basis of the optimal supportive treatment in high-risk IgAN. The evidence from this study will provide a novel, effective, and safe Chinese characteristic therapy for high-risk IgAN patients.

**Trial registration:**

ClinicalTrials.gov, NCT03418779. Registered on 18 June 2018.

## Background

IgA nephropathy (IgAN) is the most prevalent primary glomerular disease worldwide and accounts for 45% of primary glomerular disease in China, significantly contributing to the global burden of chronic kidney disease (CKD) [1, 2]. Patients with IgAN are often diagnosed at a young age, and about 30% of the patients develop end-stage renal disease (ESRD) in 10–20 years [1, 3]. Proteinuria, decreased kidney function, and hypertension at diagnosis are the independent risk factors for progression to ESRD. Among them, persistent proteinuria is the strongest predictor for developing ESRD [4–6]. According to the 2016 Oxford Classification of IgAN, mesangial or endocapillary hypercellularity, crescents, segmental glomerulosclerosis, and tubular atrophy/interstitial fibrosis are pathological indicators of poor renal outcome [7].

The Latest Kidney Disease: Improving Global Outcomes (KDIGO) Clinical Practice Guideline for Glomerulonephritis recommends angiotensin-converting enzyme inhibitors (ACEIs) or angiotensin-receptor blockers (ARBs) with uptitration to achieve a maximally tolerated dose as an initial therapy for progressive IgAN. For those patients with persistent proteinuria > 1 g per day and estimated glomerular filtration rate (eGFR) > 50 mL/min/1.73 m^2^, a 6-month course of high-dose corticosteroid therapy is suggested despite 3–6 months of optimized supportive care (ACEI, ARB, or both, and blood-pressure control). Intensive immunosuppression (corticosteroid with cyclophosphamide (CTX) or azathioprine) is reserved for patients with crescents in more than half of the glomeruli and rapidly deteriorating renal function [8]. A meta-analysis has shown that IgAN patients with more serious pathological features may be more resistant to steroid therapy than other IgAN patients according to the Oxford classification system [9].

However, the benefits of systemic immunosuppression have been questioned in recently completed trials. Conflicting results indicate the presence of severe adverse events (SAEs), which imply long-term immunosuppression must be balanced against benefits carefully [10–12]. Other general immunosuppressants are waiting for more high-quality trials to assess their safety and efficacy, including mycophenolate mofetil [13, 14], calcineurin inhibitors [15], and targeted immunosuppression regimens, like rituximab [16], targeted release formation budesonide [17], and the selective enzyme spleen tyrosine kinase inhibitor fostamatinib, which was studied in a recently completed phase II randomized controlled trial (RCT; ClinicalTrials.gov, NCT02112838),. Above all, for high-risk IgAN, safe, disease-specific therapy remains limited.

**Fig. 1:**
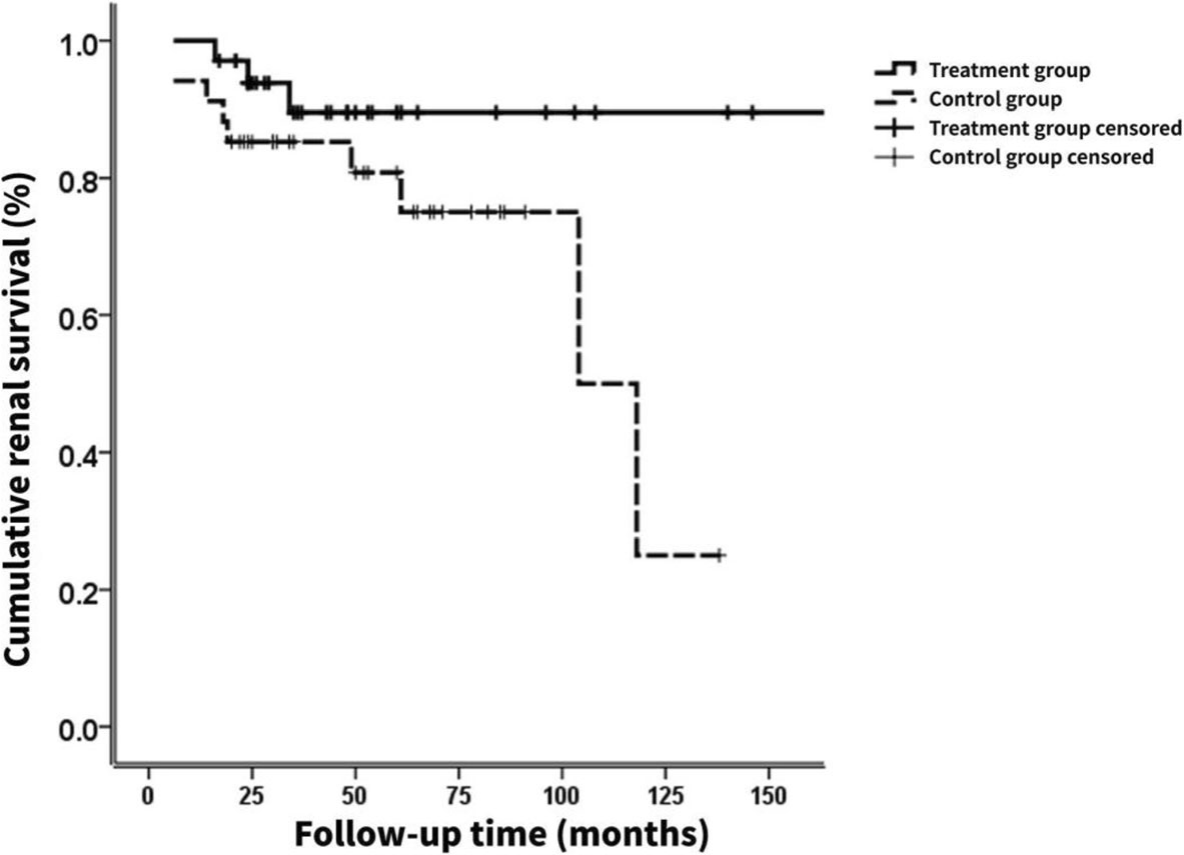
Cumulative renal survival curves

Chinese medicine is gradually accumulating evidence-based applications in CKD and has been widely approved for its positive role in prevention and treatment of IgAN [18–21]. Yi-Qi-Qing-Jie formula (YQF) was developed from the classical Chinese prescription Yupingfeng San, Yinqiao San, and Wuweixiaodu Yin, the main constituents including *Astragali radix* (HUANG QI), *atractylodes rhizome* (BAI ZHU), *Radix Saposhnikoviae* (FANG FENG), *oldenlandia diffusa* (BAI HUA SHE SHE CAO), *Rhizoma Dioscoreae Nipponicae* (CHUAN SHAN LONG), and *raw rhubarb* (DA HUANG). We conducted an ambispective cohort study [22] that matched 34 high-risk IgAN patients (UTP > 3 g/24 h and eGFR < 60 ml/min/1.73 m^2^) who received YQF combined therapy (treatment group) to 34 patients who received immunosuppression monotherapy (control group) from the Peking University First Hospital nephrology department, on the basis of renin-angiotensin system blockade (RASB). This YQF combined therapy exhibited a potential renal protective effect during the mean follow-up period of 43 months. Five patients (14.71%) developed ESRD (Fig. 1) and no SAEs were associated with the immunosuppressants. In a study by Mitsuiki et al. [23], which was similar to our treatment protocol, six patients (22%) treated with prednisolone and cyclophosphamide reached ESRD during the mean follow-up period of 66.5 months, and two patients (7.4%) suffered adverse effects of immunosuppression during treatment. However, their study did not use a standardized clinical design. Hence, we will conduct a randomized, prospective, double-blind (placebo) controlled trial to confirm that, compared with immunosuppressive therapy alone, YQF combined with immunosuppressive therapy will be superior with regard to renal function protection and reducing severe treatment-related adverse effects in patients with high-risk IgA nephropathy.

## Methods/design

### Study design

**Fig. 2:**
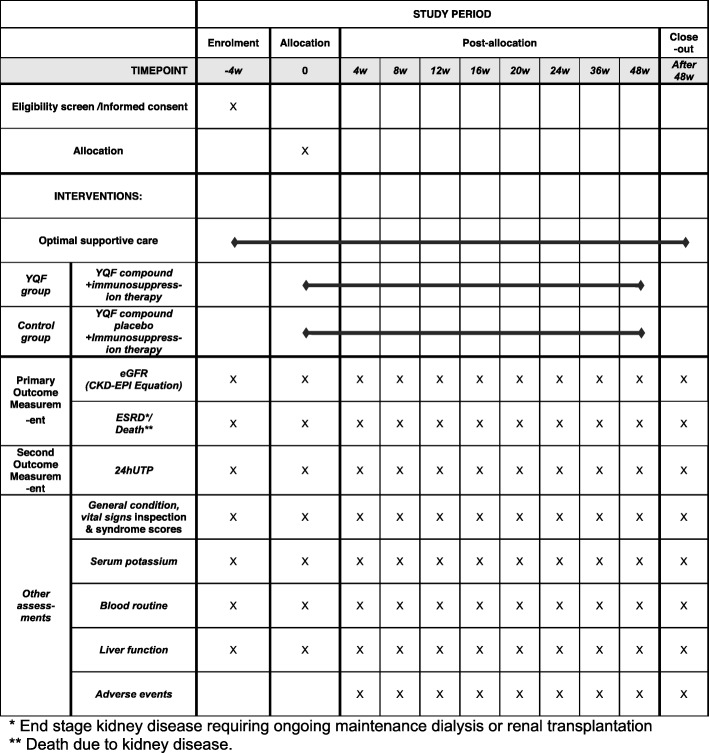
Study schedule (SPIRIT figure). * End stage kidney disease requiring ongoing maintenance dialysis or renal transplantation. ** Death due to kidney disease

This is a single-center, prospective, double-blind, placebo-controlled randomized trial. This clinical trial is reported according to the Standard Protocol Interventions: Recommendations for Interventional Trials (SPIRIT) guidelines [24] (the study schedule (SPIRIT figure) is outlined in Fig. 2, and the checklist is provided in Additional file 1).

### Setting and participants

Sixty high-risk IgAN participants will be followed up until 50% (30 of them) have a composite endpoint or have been followed for 3 years. The trial will be conducted at Guang’anmen Hospital, Beijing, China, and was approved by the Ethics Committee of Guang’anmen Hospital (approval number 2018–055-KY-01) in accordance with the Declaration of Helsinki and the principles outlined in the “Guidelines for Good Clinical Practice” from the International Conference on Harmonisation of Technical Requirements for Registration of Pharmaceuticals for Human Use Tripartite Guideline (January 1997). Recruited participants will be self-selected or referred from inpatients or outpatients and hospital-based WeChat advertisements. Potential participants will be pre-screened through WeChat, further assessed by our investigator, and given consent to participate in the study. Participation will be voluntarily and all subjects will sign informed consent.

### Objectives

Based on optimal supportive care, the trial is aiming to assess superiority with regard to renal protection and reduction of severe treatment-related adverse events of YQF combined therapy compared with immunosuppression monotherapy in high-risk IgAN.

### Rationale of high-risk IgAN

Several high-quality clinical trials [11, 12] have interpreted the concept of “high-risk” clinically and pathologically while not specifically. Following the inclusion criteria of these trials, this study will define “high-risk” as persistent heavy proteinuria (≥ 1 g/d despite intensive optimal supportive care) with impaired renal function (eGFR 15–60 ml/min/1.73 m^2^).

### Inclusion and exclusion criteria

The inclusion criteria are as follows: (1) in accordance with IgAN pathological diagnosis, renal biopsy within 6 months; (2) persistent proteinuria ≥ 1 g/d despite at least 8 weeks of optimal supportive care (maximally tolerated RAS blocker which refers to no symptomatic hypotension, no hyperkalemia, and serum creatinine (SCr) increased by not more than 30% of baseline, blood pressure control meeting targets (135/85 mmHg or lower), and dietary management (sodium intake less than 6 g/d, protein intake of 0.6–0.8 g/kg/day, and low-fat diet)); (3) eGFR 15 to 60 ml/min/1.73 m^2^, calculated with the use of CKD-EPI Creatinine Equation 2009; (4) patients who maintain regular follow-up at Guang’anmen Hospital, agree to participate, and provide informed consent (Additional file 2 shows the informed consent form in more detail).

The exclusion criteria are as follows: (1) secondary IgAN; (2) comorbidity of other primary or secondary glomerular diseases; (3) comorbidity of severe primary diseases such as cardiovascular, hepatic, cerebral, and hematopoietic system diseases or mental disorders; (4) allergy or intolerance to the experimental medication (e.g., RAS blockers, prednisolone, cyclophosphamide, YQF compound and its placebo compound); (5) contraindications to immunosuppression therapy—acute and chronic infectious diseases, malignancies, leukopenia, thrombocytopenia, gastrointestinal hemorrhage, ulcers of stomach or duodenum, post-transplantation; (6) pregnant or lactating women; (7) unwilling to participate in this study, failure to accept or tolerate Chinese medicine compound; (8) history of alcohol or drug abuse; (9) poor compliance, loss to follow-up; (10) participation in another clinical investigation.

### Randomization and masking

Subjects that are eligible to participate and have signed the informed consent will be randomly selected in 1:1 ratio after the run-in phase such that the YQF group and the control group will both include 30 patients. A central stochastic system developed by the Institute of Basic Research in Clinical Medicine, China Academy of Chinese Medical Sciences, based on SAS 9.4.3 (SAS Institute, Cary, NC, USA), will be used to perform the randomization. Independent experts will generate the randomization sequence, which will be concealed in opaque, sealed, and stapled envelopes. Participants, investigators, and all other members with clinical involvement in the trial will be blinded to the treatment assignment for the duration of the trial. Relevant personnel have clear divisions of labor and strict permission restrictions. The blinding will be removed only if a participant has severe side effects that the affected participant will be withdrawn.

### Interventions

#### Run-in period (pre-trial, 4 weeks)

Eligible participants will enter a run-in period for 4 weeks, during which they will receive an optimized basic treatment, including lifestyle management (smoking cessation, alcohol restriction, weight control, low-salt and proper protein diet), maximum tolerated dose of ACEI or ARB, blood pressure control to a target below 135/85 mmHg, control of glycated hemoglobin ≤ 7% using insulin or oral hypoglycemic agents in diabetics, reaching targets in serum uric acid (UA) (< 420 μmol/L in male, < 360 μmol/L in female) by UA-lowering medications in hyperuricemia, and stopping other Chinese medicine treatments.

#### Treatment period (in-trial, 48 weeks)

At the end of the run-in period, participants who fulfill all eligibility criteria and no exclusion criteria will be randomized to either the YQF combined immunosuppression therapy (YQF group) or matching placebo combined with immunosuppression therapy (control group) in a double-blind fashion with a total treatment period of 48 weeks. Both groups will continue their basic pre-trial treatment.

##### Immunosuppression therapy

Immunosuppression therapy comprises oral prednisolone (0.5–0.8 mg/kg/day; exact dose decided by the investigator, maximum dose not exceeding 60 mg/day) for 8 weeks, then tapered by 5–10 mg/day every 4 weeks, with a total treatment period of 24–32 weeks. Participants with persistent proteinuria ≥ 1 g/day after 8 weeks of corticosteroid monotherapy will receive 0.8–1.0 g of intravenous cyclophosphamide (CTX) every 4 weeks, total dose of not exceeding 8 g (exact dose decided by the site investigator). If severe CTX-related adverse events occur, such as alanine transaminase (ALT) exceeding the upper limit of two times, infections requiring hospitalization, granulocytes < 3.0 × 10^9^/L and platelets < 50.0 × 10^9^/L, CTX will stop being administered, symptoms will be treated, and adverse events recorded. Also, the frequency of detection will be increased to once every 2 weeks and the affected participant will be withdrawn if persistent infection or myelosuppression occurs.

##### Yi-Qi-Qing-Jie formula

YQF is obtained from Sichuan Xinlvyao Co. (Chengdu, Sichuan, China), which uses a manufacturing process complying with Chinese GMP. The compounds are blends of individual herbal extracts from YQF (consisting of *Astragalus membranaceus*, *Saposhnikovia divaricata* (*turcz.*) *Schischk*, *Flos lonicerae*, *Angelica sinensis*, *Dioscorea nipponica*, *Hedyotis diffusa Willd*, *rhubarb*, *Spatholobus suberectus*) dissolved in 150 ml boiled water and taken orally twice a day.

##### YQF placebo

The YQF placebo is obtained from Sichuan Xinlvyao Co. (Chengdu, Sichuan, China), which uses a manufacturing process complying with Chinese GMP. The major component of the placebo is malt dextrin and it comes in packaging with a similar appearance to YQF; it is also dissolved in 150 ml boiled water and taken orally twice a day.

#### Follow-up period (post-trial)

All participants will continue their previous basic treatments.

#### Follow-up assessments

Participants will be visited at regular intervals for a planned mean of at least 3 years until the end point occurs. In the run-in and treatment periods, study visits occur every 4 weeks until week 24**,** and every 12 weeks face-to-face or by telephone according to the choice of the participants and investigators until the end of the trial. Measurements will be performed at qualified laboratories during the follow-up period.

Laboratory measurements include: (1) urine tests—24 h urine total protein (UTP) measured using biuret method and albumin/creatinine ratio (ACR); (2) blood tests—serum creatinine, albumin (ALB), blood urea nitrogen (BUN), UA, blood glucose (GLU), total cholesterol (CHO), electrolytes (K, Na, Cl), triglyceride (TG), alanine transaminase (ALT), aspartate aminotransferase (AST), hemoglobin (HGB), white blood cell count (WBC), and platelets (PLT).

Body weight, appetite, excretory functions, stamina, mobility, sleep, etc. will also be monitored. Vital sign monitoring includes temperature, respiration, pulse, and blood pressure. Subjective symptoms and chronic inflammation status will be recorded by investigators on case report forms (CRFs) as syndrome scores for effectiveness assessment. Each symptom is scored on a four-point scale ranging from 0 (absent) to 3 (severe), and a total symptom score is calculated by adding together the values for all three symptoms. Similarly, we get a total chronic inflammation status score (Additional file 5).

**Fig. 3:**
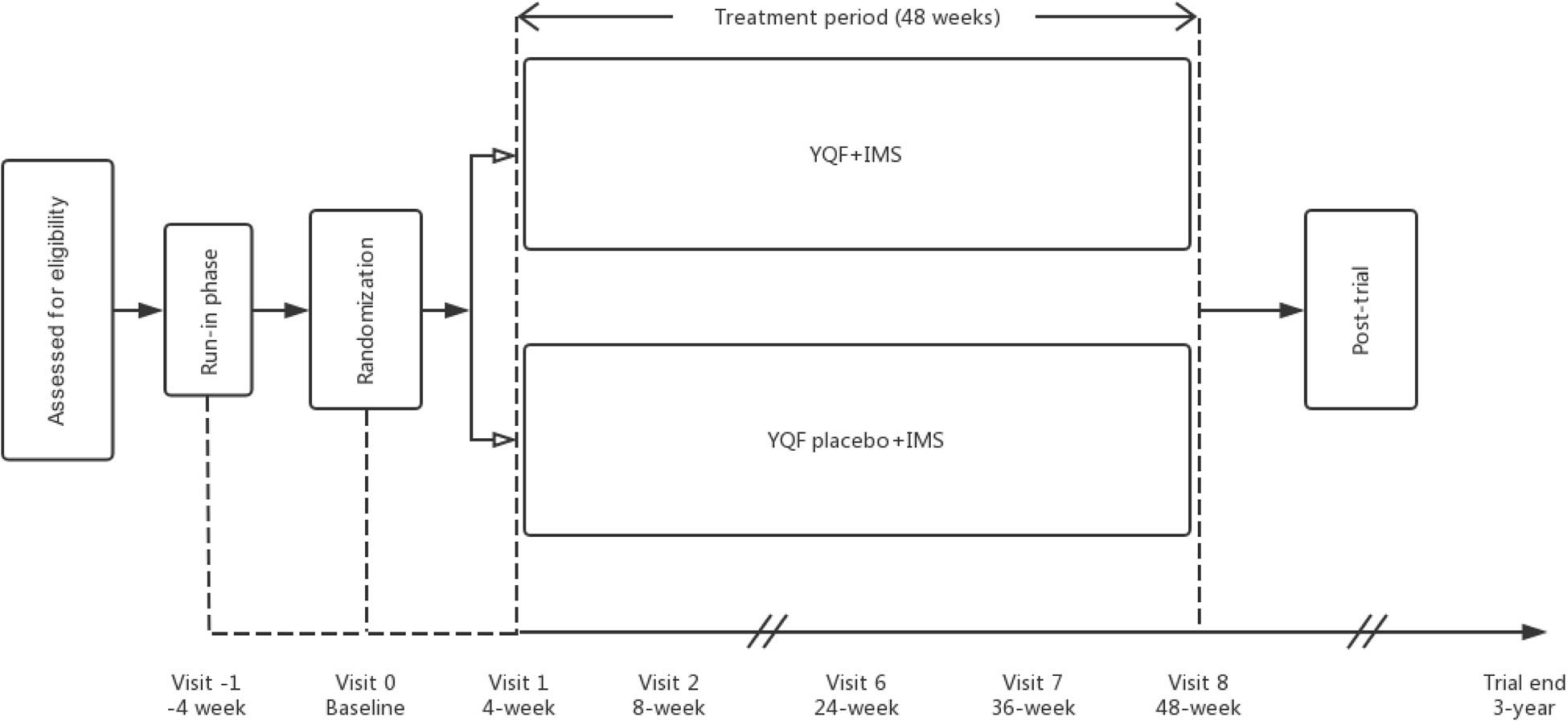
Clinical trial flow diagram

The final follow-up duration will be continued until the occurrence of the primary end point or 3 years (Fig. 3).

#### Outcome measures definition

The primary composite end point is defined as the first occurrence of 40% decrease in eGFR from baseline eGFR, progression to continuous renal replacement, or death due to renal disease.

The secondary composite end points are defined as: (1) main outcome measurement—the mean annual reduction in eGFR based on SCr (eGFR slope); (2) proteinuria remission (prescribed as proteinuria < 0.5 g/day) at weeks 24, 36, and 48 in the treatment period, and months 6, 12, 24, or 36 if possible. The remission rate of symptoms and inflammation status will be evaluated at week 48.

#### Adverse events and safety

Adverse events and SAEs will be inspected at each follow-up, including infections requiring hospitalization, thromboembolic events, hepatic dysfunction, hematopoietic disorders, fracture or osteonecrosis, and new onset diabetes. SAEs are defined according to the definitions of Good Clinical Practice (GCP) by the China Food and Drug Administration (CFDA). The researcher will report the events to the principal investigator and the ethics committee within 24 h of occurrence of the SAE, who will give a final decision on whether or not to continue the study treatment and identify appropriate support for the patients involved. Furthermore, treatment interruption due to any events and relevant measures will be recorded on the CRFs. The influence of all adverse events will be analyzed at the end of the trial.

#### Termination and withdrawal

Participants will be withdrawn from the trial in any of the following situations: (1) SAEs related to immunosuppression—severe infections (e.g., pulmonary infection requiring ventilatory support), serious granulocytopenia/thrombocytopenia (e.g., WBC < 2.0 × 10^9^/L, PLT < 20 × 10^9^/L); (2) participants or investigators fail to obey the study protocol; (3) poor compliance where less than 30% of experimental medications are taken; (4) termination requested by data and safety monitoring board, ethics committee, or study principal investigator—in the best interest of the participants, the investigator can request for termination of individual participants if the study treatment is of insignificant clinical benefit.

#### Sample size calculation

On the basis of our recent trial [22], where eGFR slope after 12 months of treatment was the primary endpoint, we define δ = (μ1 − μ2)/σ, where σ is the pooled standard deviation (for sample size calculation, see Additional file 4) and μ is the treated mean. We calculate δ = 0.994, approximately equivalent to 1.0, according to the look-up table of counts (Medical Statistics Method, PH Jin, Shanghai Medical College Press; Additional file 3), and using α = 0.05 and β = 0.1, we require a sample size of 46 participants. Assuming 25% dropout and in view of *n* = 30 as a rule of thumb for a small size clinical trial, we plan to recruit 60 patients for this study (30 patients per group).

### Statistical analysis

Investigators will fill out the CRFs when making follow-up observations by interviewing the participants. Paper CRFs will be entered into a database by those investigators in order to ensure data validity. An independent statistician will unblind and extract clean data from the final database for analysis after locking. Data analysis of treatment effect and safety follows the intention-to-treat principle. Investigators can access the final database only when both the data analysis and study are complete. The endpoint events will be described by Kaplan–Meier method and the between-group difference will be compared using log-rank test. *t*-Test will be used for scale variables, chi-square test or variance analysis will be used for binomial and nominal variables, and nonparametric test will be used for ordinal variables. The eGFR slope will be calculated as the individual slopes obtained from individual linear regressions of eGFR during the follow-up period. All analyses will be performed using SPSS software version 23.0 (IBM Analytics). Differences are considered to be statistically significant for two-sided *P* < 0.05.

#### Quality control and monitoring

A Data and Safety Monitoring Board (DSMB) will be established to monitor the performance of the overall study, ensure the safety and welfare of participants, and review quality of the data through regular meetings. The DSMB, which is independent of the present trial, consists of three members, including a clinical nephrologist, a statistician, and an ethics specialist (Additional file 6). It will assess whether participants receive good clinical care and safety concerns are interpreted and addressed appropriately. Moreover, the DSMB will make recommendations and decisions on any modification of the protocol, even termination of the study based on the interim analysis of efficacy and safety. In order to improve compliance with the protocol until completion, we will intensify patient management through one-to-one WeChat follow-up by designated researchers. Participants’ medical records (including CRFs, consent forms, etc.) will be archived securely and used only within the validity period.

Intention-to-treat analysis will be used for subjects who drop out or withdraw from the study, which includes every subject who is randomized according to randomized treatment assignment regardless of whether he/she receives the treatment or not; they will be included in the assigned group for statistical analysis of efficacy.

Last observation carried forward method will be applied in cases of missing data of longitudinal observations.

## Discussion

As IgAN is considered to be an autoimmune disease, it is logical that immunosuppression therapy may be effective [10, 25]; as the therapeutic benefits of intensive supportive care show, it is the cornerstone of treatment in IgAN. Recently completed high-profile RCTs with rigorous standardization and optimization of supportive management have focused on the outcomes of additional immunosuppression. The STOP-IgAN trial [11] showed that additional immunosuppression therapy (oral corticosteroids plus CTX and azathioprine) did not provide substantial renal protection for those with eGFR 30–59 ml/min/1.73 m^2^; this population included one death due to sepsis related to steroids. However, the trial excluded patients with 24 h UTP > 3.5 g, eGFR < 30 ml/min/1.73 m^2^, or in whom eGFR decreased more than 30% at the end of the 6-month run-in phase, which often underlies a relatively high risk for disease progression and a better response to steroids. The TESTING study [12], after a 3-month run-in phase of optimal supportive treatment, recruiting participants with 24 h UTP > 1 g and eGFR of 20–120 ml/min/1.73 m^2^, was terminated because of two deaths from infection and excess SAEs in the methylprednisolone group. Early results could not demonstrate definite treatment efficacy for steroid therapy, though they did indicate short-term potential renal benefits. Both trials highlight the safety concerns about immunosuppression and the need to seek an alternative approach which is safe and effective in high-risk IgAN.

Traditional Chinese medicine has shown promising effects regarding safety and renoprotection in some RCTs on chronic kidney diseases, for instance, *Abelmoschus manihot* [26], although it is notable that this trial excluded patients with impaired renal function (eGFR < 60 ml/min/1.73 m^2^) and nephrotic syndrome. In clinical practice, we also get patient feedback about symptomatic amelioration with respect to the application of Chinese medicine. Therefore, we are conducting TCM-WINE, a prospective, double-blind, single-placebo-controlled, randomized trial aiming to evaluate the safety and long-term outcomes with YQF herbal compound compared to immunosuppression monotherapy in treating high-risk IgAN. The results of TCM-WINE will provide clinical evidence for the efficacy and safety of YQF compound. Moreover, this trial will serve as an important step toward a new era of treatment for high-risk IgAN.

## Trial status

Recruitment for this study began on 4 July 2019 (protocol version 03, 22 November 2019). It is estimated that recruitment will be completed in August 2020.

## Supplementary information

**Additional file 1.** SPIRIT checklist.

**Additional file 2.** Informed consent form.

**Additional file 3.** Look-up table from Medical Statistics Method, PH Jin, Shanghai Medical College Press.

**Additional file 4.** Sample size calculation.

**Additional file 5.** Syndrome scale in CRF.

**Additional file 6.** DSMB members.

## Data Availability

The trial results will be published in a peer-reviewed journal and poster or oral presentations in conferences. The datasets generated or analyzed during the current study will available from the corresponding author upon reasonable request.

## Abbreviations

ACEI: Angiotensin-converting enzyme inhibitor
ACR: Albumin/creatinine ratio
ALB: Albumin
ALT: Alanine transaminase
ARB: Angiotensin-receptor blocker
AST: Aspartate aminotransferase
BUN: Blood urea nitrogen
CFDA: China Food and Drug Administration
CHO: Total cholesterol
CKD: Chronic kidney disease
CRF: Case record form
CTX: Cyclophosphamide
DSMB: Data and Safety Monitoring Board
eGFR: Estimated glomerular filtration rate
ESRD: End-stage renal disease
GCP: Good Clinical Practice
GLU: Blood glucose
HGB: Hemoglobin
IgAN: Immunoglobulin A nephropathy
KDIGO: Kidney Disease: Improving Global Outcomes
PLT: Platelets
RASB: Renin-angiotensin system blockade
RCT: Randomized controlled trial
SAE: Severe adverse event
SCr: Serum creatinine
SPIRIT: Standard Protocol Interventions: Recommendations for Interventional Trials
TG: Triglyceride
UA: Uric acid
UTP: Urine total protein
WBC: White blood cell count
YQF: Yi-Qi-Qing-Jie formula granule
