# Supplementary material for: Treatment effects of Chinese medicine (Yi-Qi-Qing-Jie herbal compound) combined with immunosuppression therapies in IgA nephropathy patients with high-risk of end-stage renal disease (TCM-WINE): study protocol for a randomized controlled trial
Source: Trials. 2020 Jan 6;21:31. doi: 10.1186/s13063-019-3989-9 (PMC6945595; doi:10.1186/s13063-019-3989-9)
Supplement: Supplementary file 2 — Additional file 2. Informed consent form. [file 13063_2019_3989_MOESM2_ESM.docx]

**Treatment effects of Chinese Medicine (****Yi-Qi-Qing-Jie Herbal Compound) combined With immunosuppression therapies in patients of IgA Nephropathy with high-risk of ESRD (TCM-WINE): Informed Consent.**

Dear patients:

We will invite you to participate in the clinical study of treatment effects of Chinese medicine Yi-Qi-Qing-Jie Formula granule (YQF) combined with immunosuppression therapies, which aims to evaluate effects and safety of YQF combined therapy compared with immunosuppression monotherapy and to provide a novel, effective and safe Chinese characteristics therapy for high-risk IgA nephropathy patients. This study is supported by the Beijing Municipal Science & Technology Commission (grant number: Z181100001718123) and the special research projects of national traditional Chinese medicine clinical research demonstration base of Guang'anmen Hospital (grant number: 2017S379). This informed consent states the purpose of the study, the study procedures, participants' rights and the responsibilities of the researchers. If you are under the age of 18, we will explain it to you and your legal guardians.

**Ⅰ. Background and objectives**

IgA nephropathy (IgAN) is the most common chronic and progressive glomerular disease in China. High-risk IgA nephropathy (persistent proteinuria>1g/day, with elevated serum creatinine) is more easily to develop end-stage renal disease, intensive monitored immunosuppression therapy is recommended for high-risk IgA nephropathy, such as corticosteroids (e.g., prednisone, prednisolone) or corticosteroid combined with immunosuppressants (e.g., cyclophosphamide, mycophenolate). An international large-scale high-profile clinical trial dominated by domestic scholars shows that immunosuppression therapy may have a lower risk of kidney failure outcome (1/3 of general treatment) but it also leads to adverse events associated with treatment, such as recurrent infection and osteoporosis. Therefore, this approach still needs more research and improvement on it.

Traditional Chinese medicine has been widely approved for its positive role in the prevention and treatment of IgA nephropathy. As the key department of the state administration of traditional Chinese medicine, nephrology department of Guang'anmen Hospital, China Academy of Chinese Medical Sciences, has put great efforts in prevention and treatment of chronic kidney disease. Following the lead of professor Xi-wen Dai, we treat high-risk IgA nephropathy using Yi-Qi-Qing-Jie Formula combined with immunosuppression therapy which exhibits a potential renal protective effect, reduces the adverse events related with immunosuppression in preliminary and improves quality of patients' life.

This study will be performed in Guang'anmen Hospital, and approximately 60 high-risk IgAN participants will be enrolled on voluntary basis and randomized to YQQJ combined group (YQQJ compound, addition to immunosuppression therapy) and immunosuppression group. Nephrologists will provide guideline recommended treatments of IgA nephropathy, including lifestyle management, blood pressure control (using renin-angiotensin system inhibitors), lipids, and uric acid management.

**Ⅱ. Research content**

The study was approved by the Ethics Committee of Guang'anmen Hospital in accordance with the Declaration of Helsinki. If you are diagnosed with biopsy-proven IgA nephropathy within 6 months, have a persistent proteinuria≥1g/d and eGFR 15 to 60ml/min/1.73m^2^, doctors will arrange you participate in the study on voluntary basis after informed consent is obtained. You will be randomized to YQF group (YQF compound dissolving in 150ml boiled water and taking it orally twice a day, addition to 0.5-0.8mg/kg/d of oral prednisolone for 8 weeks, tapered by 20% every 4 weeks for 28-32 weeks, and 0.8-1.0g of intravenous cyclophosphamide every 4 weeks for a total dose of 8g as required), or immunosuppression group (YQQJ compound placebo, addition to 0.5-0.8mg/kg/d of oral prednisolone for 8 weeks, tapered by 20% every 4 weeks for 28-32 weeks, and 0.8-1.0g of intravenous cyclophosphamide every 4 weeks for a total dose of 8g as required). Participants will be visited at regular intervals, study visits occur every 4 weeks until week 48 in treatment period, and every 12 weeks until the end of the study (3 years). Researchers will collect your general information and lab examinations results (including kidney function, liver function, blood and urine routine tests, proteinuria and urinary albumin creatinine ratio), and complete case report form at every follow-up.

The exclusion criteria are as follows: (1) secondary IgAN; (2) comorbidity of other primary or secondary glomerular diseases; (3) comorbidity of severe primary diseases such as cardiovascular, hepatic, cerebral, hematopoietic system diseases and mental disorders; (4) allergy or intolerance to the experimental medication (e.g., RAS blockers, prednisolone, cyclophosphamide, YQQJ compound and its placebo compound) ; (5) contraindications of immunosuppression therapy: acute and chronic infectious diseases, malignancies, leukopenia, thrombocytopenia, gastrointestinal hemorrhage, ulcers of stomach or duodenum, post-transplantation, and etc.; (6) pregnant or lactating women; (7) unwilling to participate in this study, failure to accept or tolerate Chinese medicine compound; (8) a history of alcohol or drug abuse; (9) poor compliance, loss to follow-up from the study.

**Ⅲ. Participation and withdrawal**

Participation is entirely voluntary. You reserve the rights to withdraw from the study at any time. Your participation or refusal of the study will not affect any rights or future interests. If you disobey the study protocol (e.g., frequent loss to follow-up) or other unspecified special circumstances, researchers may terminate your study without your consent. The ethics committee and the pharmaceutical administration may terminate your participation in the trial ahead of schedule. If you decide to withdraw from the study, inform your doctors in advance, you may be asked for last examination for securing your safety, and medical data collected before withdrawal may be adopted. In study process, researchers will terminate study according to your disease condition (e.g., treatment-related adverse events).

**Ⅳ. Benefits**

You will receive a detailed treatment instruction of the doctors, including disease monitoring and treatment guide. You can consult any question about the study by joining a wechat group started by researchers, and you may obtain lab examinations for free. We will provide with 100 yuan for transportation allowance when the follow-up ends.

**Ⅴ. Risks**

The study will not bring any extra harm to your disease treatment. The researchers will spare no efforts to avoid harm of the study to you. If adverse events caused by test drugs occur in the study, we will provide timely and proper treatment to you.

**Ⅵ. Related expenses**

This study does not interfere with your normal medical expenses (you need to pay for the related treatment, medical expenses and lab examinations). Participants will receive urine routine test and urine total protein test free of charge.

**Ⅶ. Privacy protection**

Your personal information and medical records are confidential. Only researchers, ethics committee or the authorities can inspect or supervise your medical records or personal information. Any publication of this study or data application in relative studies will not disclose your identity information. Your medical records will be kept and used only within the validity period. You may also declare refusal to offer your medical records in any other study.

**Ⅷ. Contact information**

If you have any questions about your participation in the study, please contact the researchers via WeChat or by phone (010-88001057).

If you have any questions on the rights and interests or ethics of the participants in this study, please contact the Guang'anmen Hospital Ethics Committee, at 010-88001552. Working hours: 8:00 ~ 17:00 from Monday to Friday.

**Ⅸ. What should I do now?**

You can discuss it with your families or intimate friends before participation.

Before you make decision to participate in this study, please consult it with your doctor until you fully understand.

Thank you for reading. Please keep this safe.

**Signature Page of the Informed Consent**

Name of study: Treatment effects of Chinese Medicine (Yi-Qi-Qing-Jie Herbal Compound ) combined With immunosuppression therapies in patients of IgA Nephropathy with high-risk of ESRD (TCM-WINE).

Sponsor: Guang'anmen Hospital, China Academy of Chinese Medical Sciences.

Ethical approval Number of Guang'anmen Hospital Ethics Committee: 2018-055-KY-01.

Statement of consent:

I have read the above introduction to this study, got the opportunity to discuss this study with my doctors and received satisfactory replies to all the questions I brought up.

I have known all the potential risks and benefits of participating in this study.

I know that participating is on voluntary, and confirm that I have enough time to consider about it, and I understand:

• I am able to consult my doctor for more information at any time;

• I am able to withdraw from this study at any time without any discrimination or retaliation, and the medical treatment and benefits will not be affected.

I am also clear that if I withdraw from the study, I will inform the doctor of the disease condition and complete the physical examination and physio-chemical examinations, which will be very beneficial to myself and the whole study.

I permit the researchers, the ethics committee and relative studies reviewing my medical records.

I will receive one signed and dated copy of the informed consent.

Above all, I decide to take part in this study, and I promise to follow the doctor's instructions.

Agree (signature) Disagree (signature)

Signature of the subject: ___________

Signature of legal authorized agent: ____________ Date: __________________________

phone: ___________________ Mobile: ________________________

_______________________________________________________________________________

I make sure that I have explained the details of this study to the patients, including their rights and potential benefits and risks, and the signed and dated copy of the informed consent have been given to the patients.

Phone: 010-88001057

Signature of the doctor: __________________ Date: ________________________
