# Supplementary material for: Treatment effects of Chinese medicine (Yi-Qi-Qing-Jie herbal compound) combined with immunosuppression therapies in IgA nephropathy patients with high-risk of end-stage renal disease (TCM-WINE): study protocol for a randomized controlled trial
Source: Trials. 2020 Jan 6;21:31. doi: 10.1186/s13063-019-3989-9 (PMC6945595; doi:10.1186/s13063-019-3989-9)
Supplement: Supplementary file 3 — Additional file 3. Look-up table from Medical Statistics Method, PH Jin, Shanghai Medical College Press. [file 13063_2019_3989_MOESM3_ESM.pdf]

附表 35-6 对两个样本均数的差别作统计意义检验时所需样本大小(n)

双侧检验	α=0.01					α=0.02					α=0.05					α=0.1				
单侧检验	α=0.005					α=0.01					α=0.025					α=0.05				
β=	0.01	0.05	0.1	0.2	0.5	0.01	0.05	0.1	0.2	0.5	0.01	0.05	0.1	0.2	0.5	0.01	0.05	0.1	0.2	0.5
δ/σ																				
0.05																				
0.10																				
0.15																				
0.20																				137
0.25															124					88
0.30										123					87					61
0.35					110					90					64				102	45
0.40					85					70				100	50			108	78	35
0.45				118	68				101	55			105	79	39		108	86	62	28
0.50				96	55			106	82	45		106	86	64	32		88	70	51	23
0.55			101	79	46		106	93	68	38		87	71	53	27	112	73	58	42	19
0.60		101	85	67	39		90	74	58	32	104	74	60	45	23	89	61	49	36	16
0.65		87	73	57	34	104	77	64	49	27	88	63	51	39	20	76	52	42	30	14
0.70	100	75	63	50	29	90	66	55	43	24	76	55	44	34	17	66	45	36	26	12
0.75	88	66	55	44	26	79	58	48	38	21	67	48	39	29	15	57	40	32	23	11
0.80	77	58	49	39	23	70	51	43	33	19	59	42	34	26	14	50	35	28	21	10
0.85	69	51	43	35	21	62	46	38	30	17	52	37	31	23	12	45	31	25	18	9
0.90	62	46	39	31	19	55	41	34	27	15	47	34	27	21	11	40	28	22	16	8
0.95	55	42	35	28	17	50	37	31	24	14	42	30	25	19	10	36	25	20	15	7
1.00	50	38	32	26	15	45	33	28	22	13	38	27	23	17	9	33	23	18	14	7
1.1	42	32	27	22	13	38	28	23	19	11	32	23	19	14	8	27	19	15	12	6
1.2	36	27	23	18	11	32	24	20	16	9	27	20	16	12	7	23	16	13	10	5
1.3	31	23	20	16	10	28	21	17	14	8	23	17	14	11	6	20	14	11	9	5
1.4	27	20	17	14	9	24	18	15	12	8	20	15	12	10	6	17	12	10	8	4
1.5	24	18	15	13	8	21	16	14	11	7	18	13	11	9	5	15	11	9	7	4
1.6	21	16	14	11	7	19	14	12	10	6	16	12	10	8	5	14	10	8	6	4
1.7	19	15	13	10	7	17	13	11	9	6	14	11	9	7	4	12	9	7	6	3
1.8	17	13	11	10	6	15	12	10	8	5	13	10	8	6	4	11	8	7	5	
1.9	16	12	11	9	6	14	11	9	8	5	12	9	7	6	4	10	7	6	5	
2.0	14	11	10	8	6	13	10	9	7	5	11	8	7	6	4	9	7	6	4	
2.1	13	10	9	8	5	12	9	8	7	5	10	8	6	5	3	8	6	5	4	
2.2	12	10	8	7	5	11	9	7	6	4	9	7	6	5		8	6	5	4	
2.3	11	9	8	7	5	10	8	7	6	4	9	7	6	5		7	5	5	4	
2.4	11	9	8	6	5	10	8	7	6	4	8	6	5	4		7	5	4	4	
2.5	10	8	7	6	4	9	7	6	5	4	8	6	5	4		6	5	4	3	
3.0	8	6	6	5	4	7	6	5	4	3	6	5	4	4		5	4	3		
3.5	6	5	5	4	3	6	5	4	4		5	4	4	3		4	3			
4.0	6	5	4	4		5	4	4	3		4	4	3			4				

由 扫描全能王 扫描创建
