# Supplementary material for: Treatment effects of Chinese medicine (Yi-Qi-Qing-Jie herbal compound) combined with immunosuppression therapies in IgA nephropathy patients with high-risk of end-stage renal disease (TCM-WINE): study protocol for a randomized controlled trial
Source: Trials. 2020 Jan 6;21:31. doi: 10.1186/s13063-019-3989-9 (PMC6945595; doi:10.1186/s13063-019-3989-9)
Supplement: Supplementary file 5 — Additional file 5. Syndrome scale in CRF. [file 13063_2019_3989_MOESM5_ESM.docx]

**Each symptom is scored 0, 1, 2, and 3 for absent, mild, moderate, and severe, respectively**

| Symptom | Grade | Absent | Mild | Moderate | Severe |
| --- | --- | --- | --- | --- | --- |
| Lassitude, and weary limbs |  |  | I can only take on light manual labor, and feel fatigue occasionally | I get tired very quickly, and can’t do much during the day | I have problems to start things, feeling constantly exhausted both physically and mentally |
| Shortness of breath |  |  | I get tired when talking too much | I get short of breath and talk less | I feel too exhausted to make a sound |
| Predisposition to having common cold |  | Less than once per year | 2-3 times per year | 4-6 times per year | More than 6 times per year |
| Chronic Inflammation Status |  |  |  |  |  |
| Rhinitis |  | Less than once per year | 2-3 times per year | 4-6 times per year | More than 6 times per year |
| Pharyngitis |  | Less than once per year | 2-3 times per year | 4-6 times per year | More than 6 times per year |
| Tympanitis |  | Less than once per year | 2-3 times per year | 4-6 times per year | More than 6 times per year |
| Chronic bronchitis |  | Less than once per year | 2-3 times per year | 4-6 times per year | More than 6 times per year |
| Chronic enteritis |  | Less than once per year | 2-3 times per year | 4-6 times per year | More than 6 times per year |
| Urinary infection |  | Less than once per year | 2-3 times per year | 4-6 times per year | More than 6 times per year |
| Prostatitis |  | Less than once per year | 2-3 times per year | 4-6 times per year | More than 6 times per year |
| Pelvic inflammation |  | Less than once per year | 2-3 times per year | 4-6 times per year | More than 6 times per year |
| Erysipelas on lower limbs |  | Less than once per year | 2-3 times per year | 4-6 times per year | More than 6 times per year |
| Defecation frequency |  | | | | |
| Tongue appearance and pulse feelings |  | | | | |
