# Supplementary material for: Treatment effects of Chinese medicine (Yi-Qi-Qing-Jie herbal compound) combined with immunosuppression therapies in IgA nephropathy patients with high-risk of end-stage renal disease (TCM-WINE): study protocol for a randomized controlled trial
Source: Trials. 2020 Jan 6;21:31. doi: 10.1186/s13063-019-3989-9 (PMC6945595; doi:10.1186/s13063-019-3989-9)
Supplement: Supplementary file 6 — Additional file 6. DSMB members. [file 13063_2019_3989_MOESM6_ESM.docx]

**Data and Safety Monitoring Board members**

Yongli Zhan, Ph.D, Professor, Clinical Nephrologist

Renal Division, Guang’anmen Hospital, China Academy of Chinese Medical Sciences, Beijing, China

E-mail: [zhanyongli88@sina.com](mailto:zhanyongli88@sina.com)

Fengmei Lian, Ph.D, Researcher, Clinical Pharmacologist and Statistician

Endocrinology Division, Guang’anmen Hospital, China Academy of Chinese Medical Sciences, Beijing, China

E-mail: lfm565@sohu,com

Jie Qiao, Ph.D, Attending Physician, Ethical Specialist

Ethics Committee of Guang’anmen Hospital, China Academy of Chinese Medical Sciences, Beijing, China

E-mail: qjie00@126.com
